# Clinical features and outcomes of adult primary retroperitoneal lymphangioma: insights from a high-volume sarcoma center

**DOI:** 10.3389/fsurg.2026.1760065

**Published:** 2026-02-12

**Authors:** Wenjie Li, Mengmeng Xiao, Lei Liu, Haining Zheng, Haicheng Gao, Boyuan Zou, Dehu Lu, Mei Huang, Chengli Miao

**Affiliations:** 1 Department of Retroperitoneal Tumor Surgery, Peking University International Hospital, Beijing, China; 2 Department of General Surgery, Peking University People’s Hospital, Beijing, China; 3 Department of Pathology, Peking University International Hospital, Beijing, China; 4 Department of Ultrasound, Peking University International Hospital, Beijing, China

**Keywords:** adult primary retroperitoneal lymphangioma, imaging manifestation, laparoscopic surgery, prognosis, surgical treatment

## Abstract

**Background:**

Adult primary retroperitoneal lymphangioma (RPL) is an exceptionally rare benign vascular malformation. This study aims to elucidate the clinical presentation, imaging characteristics, management strategies, and long-term outcomes of this condition.

**Methods:**

We conducted a retrospective analysis of our prospectively maintained retroperitoneal tumor database at Peking University International Hospital (2014–2024). Inclusion criteria comprised adults (≥18 years) undergoing initial surgical resection with pathological RPL confirmation. Patients with other malignancies or recurrent disease were excluded. Comprehensive follow-up was performed to assess outcomes.

**Results:**

Among 28 enrolled patients (13 males, 15 females; median age 31 years), clinical presentation included incidental discovery (42.9%), abdominal discomfort (42.9%), lumbago (10.7%), and lower limb pain (3.6%). Preoperative imaging assessment was performed using Ultrasonography (US) (82.1%), computed tomography (CT, 100.0%), and magnetic resonance imaging (MRI, 14.3%). Characteristic CT findings typically included thin-walled multiloculated cystic masses with septal enhancement. All patients achieved R0/R1 resection, with 18 open and 10 laparoscopic procedures (2 conversions). Major complications occurred in 10.7% of cases (lymphatic leakage: 2; pancreatic fistula: 1), all resolving with appropriate management. Histopathological and immunohistochemical analysis confirmed lymphatic differentiation (D2-40: 100%; CD31: 89.3%; CD34: 84.6%). During median follow-up of 76 months, no recurrences or disease-specific mortality were observed.

**Conclusions:**

RPL represents a rare benign tumor frequently presenting with nonspecific symptoms. Complete surgical resection demonstrates excellent safety and long-term efficacy, with individualized approach selection based on tumor characteristics. Our findings from this substantial Chinese adult cohort provide valuable insights for managing this uncommon condition.

## 1 Introduction

Lymphangiomas are rare, benign vascular malformations believed to result from the ectasia or aberrant development of lymphatic vessels (1). Although approximately 95% of cases occur in pediatric patients, primarily involving the head and neck, axillary, and mediastinal regions, presentation in adults is uncommon and may occur in diverse anatomical sites. Among these, retroperitoneal lymphangioma represents a particularly rare subset, accounting for only about 1% of all cases (2). Several factors—such as trauma, inflammation, iatrogenic injury, or fibrosis—have been proposed as potential triggers for the development of lymphangioma in adulthood, though these hypotheses await validation through future scientific studies (3).

Clinically, retroperitoneal lymphangiomas often present with non-specific symptoms and are frequently detected incidentally during imaging performed for unrelated conditions (4). Definitive diagnosis relies on histopathological examination, and the prognosis is generally favorable. Nevertheless, there remains a lack of high-quality evidence and consensus regarding the optimal management of this condition, with current understanding largely derived from small, isolated case series. Therefore, this study aims to analyze the clinical characteristics, surgical management, and long-term outcomes in a single-institution cohort of patients who underwent surgery for adult primary retroperitoneal lymphangioma, and to summarize the associated clinicopathological features and treatment experience.

## 2 Materials and methods

### 2.1 Patient selection

A retrospective review was conducted on consecutive adult patients who underwent surgical resection for retroperitoneal lymphangioma at Peking University International Hospital between January 1, 2014, and December 31, 2024. Inclusion criteria were: (1) age 18 years or older; (2) initial surgical resection; and (3) definitive pathological confirmation of retroperitoneal lymphangioma. Patients with a history of other malignancies or recurrent lesions were excluded. Based on these criteria, a total of 28 patients were included in the final analysis.

### 2.2 Data collection and statistical analysis

Data were collected from medical records and our institution's prospectively maintained retroperitoneal tumor database. The extracted information included patient demographics (gender, age), body mass index (BMI), clinical symptoms, preoperative laboratory results and radiological lesion size. Surgical details such as approach, operative time, estimated blood loss, duration of postoperative stay, and postoperative complications were also documented.

Patient follow-up was conducted routinely via telephone or clinical visits, with the final follow-up date set to August 1, 2025. The primary endpoint was defined as disease recurrence or disease-related mortality. Survival analysis was performed based on the occurrence of these endpoint events.

Categorical variables are presented as numbers and percentages. Continuous variables are expressed as mean ± standard deviation (SD) or median with interquartile range (IQR) or range, as appropriate based on normality testing. All statistical analyses were performed using SPSS version 22.0 (IBM Corp., Chicago, IL, USA).

### 2.3 Ethics approval and informed consent

This study was approved by the Institutional Review Board of Peking University International Hospital. All procedures were performed in accordance with the ethical standards of the institutional and national research committees and the 1964 Declaration of Helsinki and its later amendments (currently the 2013 Fortaleza version). Written informed consent was obtained from all individual participants or their legally authorized representatives prior to inclusion in the study.

## 3 Results

### 3.1 Clinical features

The basic clinical characteristics of the 28 adult retroperitoneal lymphangioma patients (13 males, 15 females) are summarized in Table 1. The median age was 31 years (range, 18–67 years). The main reasons for the patient's visit were physical examination and abdominal pain (24/28). Routine laboratory tests, including blood count, serum biochemistry, and tumor markers, were generally normal preoperatively. The detailed preoperative nutritional indices are presented separately in Table 2.

**Table 1:** Demographic characteristics and chief complaints of the patients (*n* = 28).

Variables	N	% or range
Total	28	(100)
Gender (male)	13	(46.4)
Median age at diagnosis (years, range)	–	31 (range: 18–67)
Chief complaints		
Physical examinations	12	(42.9)
Abdominal discomfort	12	(42.9)
Lumbago	3	(10.7)
Lower limb pain	1	(3.6)

**Table 2:** Preoperative nutritional parameters of the patients (mean ± SD).

Nutrition index	Unit	Value
Haemoglobin	g/L	132 ± 6.3
Albumin	g/L	39.1 ± 2.2
BMI	kg/m^2^	21.7 ± 4.1

### 3.2 Imaging examination

**Figure 1:**
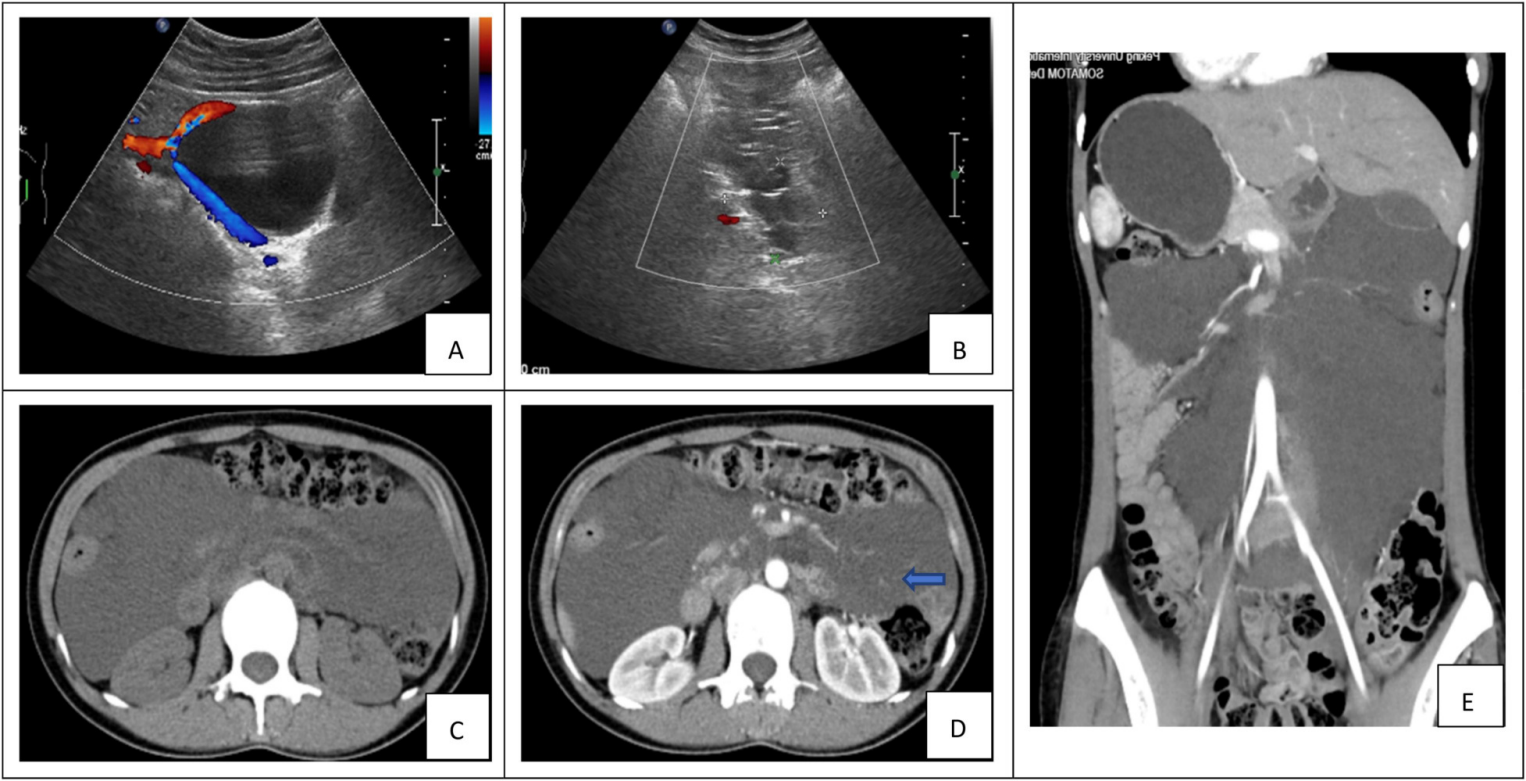
**(A–E)** Preoperative imaging of retroperitoneal lymphangioma. **(A,B)** Ultrasonography. **(A)** A well-circumscribed, oval, anechoic cystic mass is observed at the angle between the left retroperitoneum and the iliac vessels, demonstrating no internal vascular flow on Doppler imaging. **(B)** Image of a right retroperitoneal lesion reveals multiple thin-walled cystic spaces without calcification. **(C–E)** Computed Tomography (CT). **(C)** Axial plain CT scan demonstrates an irregular fluid-attenuation mass with internal septations and partially defined borders. **(D)** Contrast-enhanced axial CT shows mild enhancement of the septa (blue arrow). **(E)** Coronal reformatted CT reveals a large cystic lesion causing mass effect on adjacent organs.

Preoperative imaging evaluations among the cohort included ultrasonography (*n* = 23), contrast-enhanced computed tomography (CT; *n* = 28), and contrast-enhanced magnetic resonance imaging (MRI; all performed at outside institutions, *n* = 4). Ultrasonography typically reveals cystic lesions characterized by multiple thin septa and a honeycomb or cobweb-like echogenic pattern, which assists in distinguishing lymphatic malformations from other pathologies (Figures 1A,B). On CT, these lesions typically present as well-demarcated cystic masses with smooth walls and irregular contours. The internal density is homogeneous and approximates that of water (approximately 5–15 HU). In cases of hemorrhage or infection, the density may become heterogeneously elevated, whereas the presence of chylous content can result in negative attenuation values. Contrast-enhanced CT typically demonstrates no enhancement within the cystic fluid; however, mild enhancement may be observed along the septa or cyst wall. Larger cysts often induce a “remodeling” effect on adjacent anatomical structures (Figures 1C–E). MRI offers superior soft tissue contrast, exhibiting low to intermediate signal intensity on T1-weighted images and high signal intensity on T2-weighted images. The multiplanar capability and high contrast resolution of MRI allow precise delineation of dilated lymphatic channels, highlighting its diagnostic utility.

### 3.3 Surgical details and outcomes

All patients were hospitalized for evaluation of suspected retroperitoneal masses. The goal of the surgical approach was to achieve complete excision with dissection of surrounding tissues to obtain negative margins. Of these, eighteen patients underwent conventional open surgery, while ten underwent laparoscopic procedures. Of the latter, two required conversion to open surgery: one due to obliterated anatomical planes resulting from severe adhesions secondary to previous cholecystectomy, and the other due to uncontrolled hemorrhage encountered during dissection of a large (13 cm) tumor located posterior to the pancreas. The operative time was 150 min [interquartile range (IQR), 120–240 min]. Estimated intraoperative blood loss ranged from 50 to 400 mL, with a median of 100 mL. Three patients experienced major postoperative complications, all of whom recovered well following appropriate management. All patients were discharged successfully with no perioperative mortality or readmission within 30 days after surgery. The mean postoperative hospital stay was 12.3 ± 3.8 days. Detailed characteristics of the tumors and relevant surgical parameters are provided in Table 3.

**Table 3:** Clinicopathological and surgical characteristics of patients with retroperitoneal lymphangioma (*n* = 28).

Variables	*N*	% or range
Total	28	(100)
Surgical approaches		
Traditional open resection	18	(64.3)
Laparoscopic surgery	10	(35.7)
Conversion to open surgery	2	(7.1)
Tumor location		
Left retroperitoneal	17	(60.7)
Right retroperitoneal	11	(39.3)
Tumor classification		
Cystic lymphangioma	20	(71.4)
Cavernous lymphangioma	8	(28.6)
Capillary lymphangioma	0	(0)
Single room/multi room		
Single room	7	(25.0)
Multi room	21	(75.0)
Tumor size (cm)		
<8	6	(21.4)
≥8	22	(78.6)
OPT (min)		(120–240)
IBL (mL)		(50–400)
Resection margin		
R0	22	(78.6)
R1	6	(21.4)
R2	0	(0)
Combined resection of organs	0	(0)
Complication	3	(10.7)
Lymphatic leakage	2	(7.1)
Pancreatic fistula	1	(3.6)
Mortality	0	(0)

### 3.4 Histopathological outcomes

**Figure 2:**
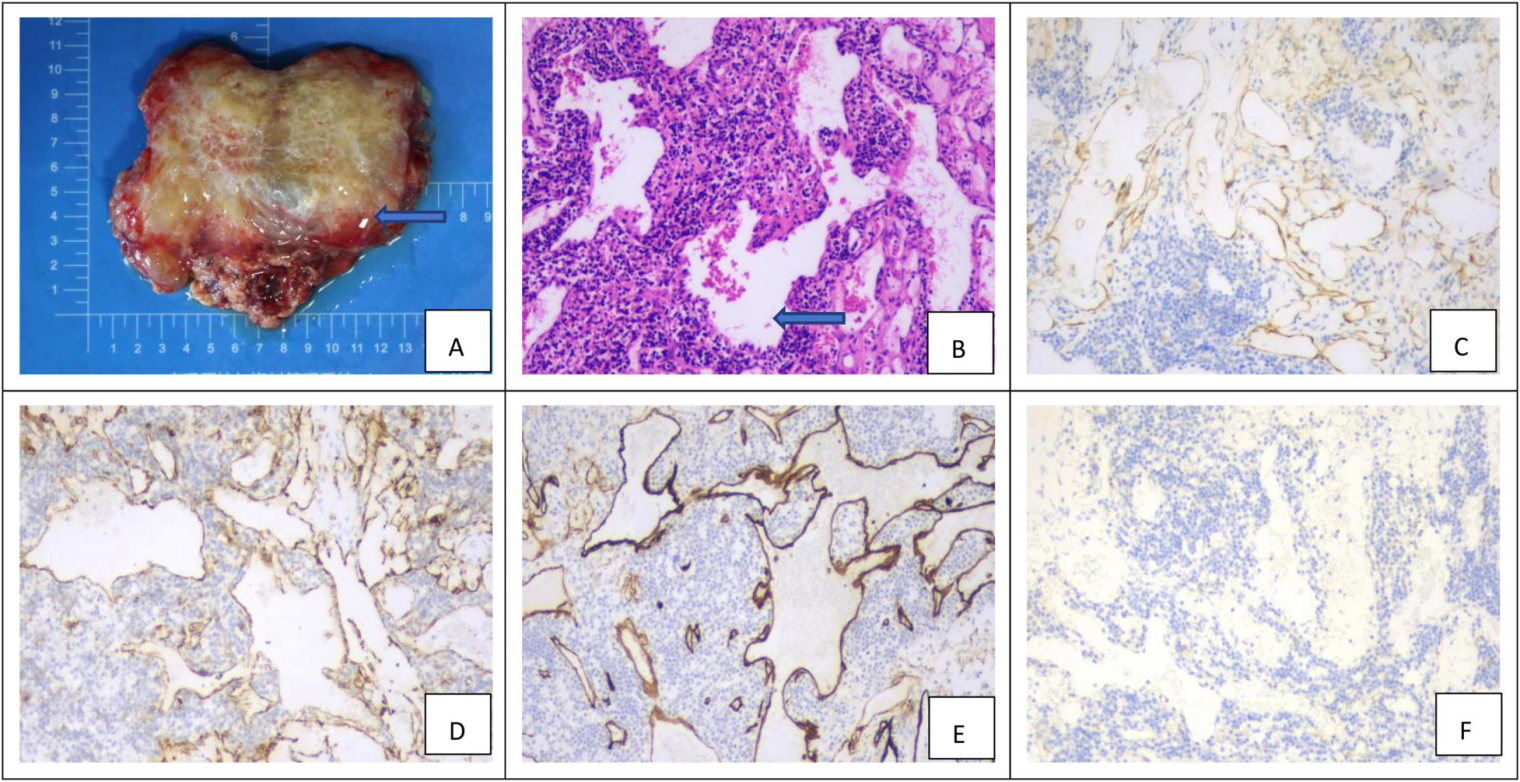
Histopathological and immunohistochemical characteristics of retroperitoneal lymphangioma. **(A)** Gross morphology of the resected multicystic lesion. The specimen comprises multiple thin-walled cysts of varying size, containing clear serous or chylous fluid (blue arrow). **(B)** Histological features on hematoxylin and eosin (H&E) staining (×100). Variably dilated lymphatic channels are lined by a single layer of flattened endothelial cells and embedded within a fibrovascular stroma (blue arrow). **(C)**Strong D2-40 (podoplanin) positivity confirms lymphatic endothelial differentiation. **(D,E)** Concomitant expression of the general endothelial markers CD31 and CD34 supports the vascular nature of the lesion. **(F)** Negativity for cytokeratin effectively excludes an epithelial origin, completing a profile diagnostic of lymphangioma (original magnification ×100 for all).

Definitive diagnosis of retroperitoneal lymphangioma (RPL) was established through histopathological evaluation. Gross examination of resected specimens revealed well-circumscribed, multiloculated cystic masses with thin and translucent walls. The cysts varied in size and contained clear serous fluid or, in some cases, chylous fluid, with generally smooth internal surfaces (Figure 2A).

Microscopic analysis showed variably dilated lymphatic channels lined by a single layer of flattened endothelial cells without atypia or significant mitotic activity (Figure 2B). These structures were supported by a fibrovascular stroma that occasionally contained scattered lymphocytes and lymphoid aggregates. The lumina were predominantly empty or filled with eosinophilic proteinaceous material.

Immunohistochemical staining confirmed the lymphatic lineage of the lesions and assisted in differential diagnosis. The endothelial cells exhibited strong cytoplasmic expression of D2-40 (Podoplanin) (Figure 2C), confirming lymphatic differentiation. Positive staining was also observed for the vascular markers CD31 (Figure 2D) and CD34 (Figure 2E). In contrast, all lesions were negative for cytokeratin (Figure 2F), effectively excluding an epithelial origin. The Ki-67 proliferation index was low (<2%), corroborating the benign nature of the lesions. Detailed results are summarized in Table 4.

**Table 4:** Immunohistochemical staining profiles in 28 patients with RPL.

Antigen	*N* of positive	Positive rate (%)
D2-40	28/28	(100)
CD31	25/28	(89.3)
CD34	11/13	(84.6)
CK	0/7	(0)
Ki-67(小于%2)	28/28	(100)

### 3.5 Follow-up results

All patients were routinely followed up via telephone interviews or clinic visits. Two patients were lost to follow-up. The median follow-up time was 76 months (range: 13–124 months). Encouragingly, all evaluated patients remained alive with a good quality of life throughout the follow-up period, and no disease recurrence was observed. Due to the absence of endpoint events, survival analysis was not performed.

## 4 Discussion

### 4.1 Epidemiological and clinical characteristics

Lymphangioma represents an uncommon benign vascular malformation of the lymphatic system, initially documented by Koch in 1913 (5). The estimated incidence ranges between 1 in 2,000 to 4,000 live births without significant gender predilection (6). Our cohort's gender distribution (13 males and 15 females) aligns with this established pattern, demonstrating nearly equal representation. While lymphangiomas can theoretically manifest at any age, their clinical presentation follows a distinct chronological pattern: approximately 50% are identified at birth, and up to 90% become apparent before two years of age, strongly supporting a congenital origin (7, 8). Adult presentation remains relatively uncommon, constituting a minority of cases.

Anatomically, while the majority of lymphangiomas (over 90%) occur in the head and neck region, primarily involving cutaneous and subcutaneous tissues, retroperitoneal localization represents a rare entity accounting for less than 5% of all cases (2). This category encompasses lesions arising in the retroperitoneal space, peritoneal cavity, and mediastinum. The spacious and anatomically complex nature of the retroperitoneal compartment allows these masses to remain clinically silent until they achieve substantial dimensions. Consequently, patients typically present with large masses at diagnosis, frequently accompanied by non-specific symptoms including abdominal discomfort, lumbago, or lower limb pain (9). In advanced cases, retroperitoneal lymphangiomas (RLs) may precipitate acute abdominal emergencies secondary to complications such as bowel obstruction, hemorrhage, or infection (10, 11). To our knowledge, the current investigation represents the first systematic documentation and largest reported Chinese adult cohort addressing this rare clinical entity.

### 4.2 Comprehensive diagnostic evaluation

The diagnostic workup for retroperitoneal lymphangioma (RPL) relies on a multimodality imaging strategy. Ultrasonography, as an accessible and non-invasive tool, is valuable for initial screening, typically revealing a thin-walled cystic lesion with well-defined margins, internal anechoic or hypoechoic content, and absent vascularity on color Doppler. The presence of multiple thin septa forming a honeycomb or cobweb-like architecture is highly suggestive of lymphatic malformation (12).

For comprehensive preoperative evaluation, CT and MRI provide superior diagnostic information (13). CT generally demonstrates a thin-walled, multiloculated cystic mass with fluid-attenuation and well-defined borders. Post-contrast images show mild septal and wall enhancement without internal uptake; calcification is uncommon (14). MRI excels in soft tissue resolution, typically displaying T1 hypointensity and T2 hyperintensity, with enhancement confined to the septa and wall. Both modalities delineate smooth contours and homogeneous non-enhancing content (6, 13). MRI is particularly useful for clarifying anatomical origin and spatial relationships with adjacent vital structures; however, in our institution, CT was often deemed sufficient for diagnosis and surgical planning once characteristic features were identified, which explains why none of the patients underwent MRI locally.

The differential diagnoses of RPL include teratoma, ovarian cyst, mucinous cystadenoma, pancreatic pseudocyst, cystic mesothelioma, Müllerian duct cyst, epidermoid cyst, and organized hematoma (15). Although imaging findings can overlap, recognition of typical RPL patterns in the appropriate clinical context aids in narrowing the differential. Beyond diagnosis, CT and MRI play an essential role in surgical planning by precisely localizing the lesion, assessing its volume, and delineating anatomical relationships. MRI is also sensitive for detecting intracystic hemorrhage (16, 17).

Despite advances in imaging, histopathological examination remains the gold standard for definitive diagnosis. Microscopic evaluation shows abnormally dilated lymphatic channels lined by flattened endothelial cells within a thin connective tissue stroma. Immunohistochemical findings in our cohort supported lymphatic differentiation: positive for D2-40 (28/28, 100%), CD31 (25/28, 89.3%), and CD34 (11/13, 84.6%), and negative for cytokeratin (0/7, 0%). The low Ki-67 index (<2% in all cases) further corroborates the benign nature of these lesions (6, 18). Thus, while advanced imaging strongly suggests the diagnosis, histopathology with immunohistochemical profiling remains definitive for RPL.

### 4.3 Therapeutic strategies and surgical technical considerations

Complete surgical resection with R0/R1 margins is the cornerstone of management for retroperitoneal lymphangioma (RPL) and is associated with favorable long-term outcomes (4). In the present cohort, all 28 patients underwent curative resection as primary treatment without requiring adjunctive therapy. The choice of surgical approach was individualized based on comprehensive preoperative evaluation (19). At our institution, laparoscopic resection was considered in cases with the following features: (1) well-circumscribed tumor borders on cross-sectional imaging; (2) predominantly cystic composition, which remains amenable to a minimally invasive approach even in larger masses; and (3) a clear plane of separation from major retroperitoneal vessels. Conversely, experience from our two converted cases highlights conditions where laparoscopy is less advisable or warrants a low threshold for conversion to open surgery, including significant adhesions from previous abdominal procedures that obscure anatomical planes, and tumors intimately adherent to major vascular structures with a high risk of uncontrollable hemorrhage.

Successful resection of RPL requires meticulous technique tailored to the complex retroperitoneal anatomy. Essential technical principles include adequate exposure, dissection along avascular planes between the tumor capsule and adjacent structures under direct vision, early vascular control, and secure ligation of lymphatic pedicles to prevent postoperative complications such as chylous leakage (20). The intraoperative observation of chylous content should prompt systematic ligation of all communicating lymphatic channels (21). Importantly, the inability to establish or maintain a clear dissection plane, or the occurrence of substantial hemorrhage that is difficult to control laparoscopically, should lead to timely conversion to an open procedure. Such a decision reflects prudent surgical judgment aimed at ensuring patient safety and achieving complete resection, rather than representing a procedural failure.

Laparoscopic surgery is consistent with enhanced recovery after surgery (ERAS) principles, offering benefits such as minimal invasiveness, reduced postoperative pain, and faster convalescence (19). However, the anatomical complexity of the retroperitoneum presents considerable technical challenges, as evidenced by a non-negligible conversion rate and the potential for vascular or visceral injury (3, 22). Therefore, laparoscopic resection of RPL should ideally be performed in high-volume referral centers with specialized expertise in advanced minimally invasive surgery.

For patients deemed at high surgical risk, non-surgical therapeutic options have been explored, including ultrasound- or CT-guided aspiration and injection sclerotherapy using agents such as alcohol or acetic acid (23, 24). While such non-surgical modalities may hold promise, their application remains largely based on isolated case reports, such as that by Park et al., in which initial ethanol ablation failed but subsequent acetic acid sclerotherapy demonstrated success (25). Therefore, these techniques should be applied with caution, and further studies are required to evaluate their efficacy and standardization in the management of RPL.

### 4.4 Postoperative outcomes and complication management

Both open and laparoscopic approaches demonstrated acceptable safety profiles, with postoperative complications occurring in 3 patients (10.7%). The most clinically significant complication was chylous ascites, which developed in two patients following resumption of oral intake. These cases presented with characteristic milky or yellowish-white drain output ranging from 300 to 600 mL daily. Both instances were successfully managed conservatively through a structured protocol involving fasting, tailored total parenteral nutrition (TPN), and subsequent gradual transition to a low-fat diet once drainage characteristics normalized, resulting in complete resolution. One patient developed a postoperative pancreatic fistula, which was effectively controlled with adequate drainage and targeted antibiotic therapy.

Notably, no tumor recurrence or disease-related mortality was observed during the follow-up period. Our institutional preference for resection with anatomically defined margins likely contributed to these favorable outcomes, underscoring the importance of complete excision even in the absence of definitive preoperative diagnosis.

### 4.5 Follow-up protocol and future directions

Currently, no universally accepted follow-up protocol exists for resected RPL. Based on available literature and institutional experience, we recommend cross-sectional imaging (CT or MRI) every 6 months for the first 3 postoperative years, followed by annual studies thereafter. Future prospective, multi-institutional studies with extended follow-up durations are warranted to validate the impact of surgical technique on long-term outcomes and refine evidence-based surveillance strategies for this rare clinical entity.

This systematic evaluation of a substantial patient cohort contributes meaningful evidence to the limited literature on RPL management, particularly within the Chinese adult population. Our findings support complete surgical resection as the treatment of choice, with appropriate technique selection based on individual patient and tumor characteristics.

## 5 Conclusions

In summary, this study affirms that complete surgical resection is a safe and effective treatment for retroperitoneal lymphangioma (RPL), resulting in excellent long-term outcomes with minimal recurrence. Preoperative diagnosis remains challenging due to nonspecific clinical presentation; thus, a combination of clinical suspicion and characteristic CT/MRI findings is essential for initial evaluation, while histopathology supported by immunohistochemical markers (e.g., D2-40) constitutes the diagnostic gold standard.

The selection of surgical approach—open or laparoscopic—should be individualized based on tumor features and surgical expertise. Although laparoscopic resection aligns with ERAS principles and promotes faster recovery, it is best reserved for carefully selected cases in specialized centers owing to the anatomical complexity of the retroperitoneum. Meticulous lymphatic pedicle ligation is crucial to preventing postoperative complications such as chylous ascites. Future multi-institutional studies with larger cohorts and extended follow-up are warranted to establish standardized, evidence-based management guidelines for this rare condition.

## Data Availability

The raw data supporting the conclusions of this article will be made available by the authors, without undue reservation.
